# Water Conservation in Orthopaedic Surgical Scrubbing: A Comparative Analysis of Water Consumption in Standard and Modified Surgical Scrubbing Techniques

**DOI:** 10.7759/cureus.99519

**Published:** 2025-12-18

**Authors:** Aaisha Shahbaz, Yash Dinesh, Kamen S Dosanjh, Sohail Muzammil, Fahad Imami, Rohma Shahbaz, Mohamed Messwi, Deepa Bose

**Affiliations:** 1 Trauma and Orthopaedic Surgery, University Hospitals Birmingham NHS Foundation Trust, Birmingham, GBR; 2 Trauma and Orthopaedic Surgery, College of Medicine and Health, University of Birmingham, Birmingham, GBR; 3 Orthopaedic Surgery, Doctors Hospital and Medical Center, Lahore, PAK; 4 General Surgery, Pakistan Air Force Hospitals, Islamabad, PAK; 5 General Surgery, Combined Military Hospital Multan, Multan, PAK

**Keywords:** healthcare sustainability, orthopaedic surgery, surgical scrub, water consumption, water reduction

## Abstract

Background

Surgical hand scrubbing plays a vital role in preventing surgical site infections. The current guidelines recommend a scrub that lasts between two and five minutes. This, however, poses a challenge where the traditional continuous flow scrub method consumes around 20 litres of water. With water scarcity quickly becoming a global issue, this highlights the importance of finding more efficient scrubbing techniques.

Objective

A comparison of the duration of scrubbing and water consumed between the standard continuous flow technique and a modified tap on/off technique, to determine if significant water savings can be achieved without compromising scrub quality.

Methods

This study was carried out in the trauma and orthopaedic department at Queen Elizabeth Hospital, Birmingham, UK. Thirty-four healthcare professionals participated by performing both standard and modified scrub techniques. To measure the amount of water, a 25-litre container along with a weighing method was used. Stopwatches were used to track the scrub duration and the time the tap was actively running.

Results

The modified scrubbing technique led to reduced water use, as average staff members used 5.6 ± 1.78 litres (mean ± SD). In the standard technique, the median water use was 16.2 L (interquartile range (IQR) 6.15). The mean saving of 11.34 ± 5.57 litres (mean ± SD) was statistically significant (p < 0.0001). Scrub times were slightly longer with the modified technique, showing a mean increase of 14.96 ± 44.30 seconds (mean ± SD), but this did not reach a statistically significant difference.

Conclusion

The modified scrubbing method delivers significant water savings without compromising the duration of the process. This makes it a practical and sustainable option, while also supporting the NHS's commitment to the environment.

## Introduction

Surgical scrubbing is the crucial process of methodically cleaning hands before surgery to remove as many microorganisms as possible and therefore reduce the incidence of surgical site infections [1,2]. It aims to eliminate the transient and reduce the resident flora. The usage of chlorhexidine or povidone-iodine scrubs initially reduces bacterial counts by 70-80%, achieving 99% on repeated application [3]. Therefore, the incidence of surgical site infections is significantly reduced.

The total duration of an adequate surgical scrub is thoroughly researched, and the New England Journal of Medicine provides detailed guidance on a five-minute surgical scrub technique; they acknowledge that a five-minute scrub reduces bacterial counts as effectively as a 10-minute scrub and that a two- to three-minute scrub reduces bacterial counts acceptably [4]. The WHO also recommends that surgical hand antisepsis should last the length of time recommended by the manufacturer, typically two to five minutes [5]. The WHO also notes that one surgical scrub with traditional agents uses approximately 20 litres of warm water [2]. The amount of water used by operating theatres is an increasing concern. In developing countries, the cost of water is rising due to increased scarcity of water, and it has resulted in the cancellation of operations due to a lack of water. One report from Nigeria showed that 5.1% of emergency operations were delayed due to a lack of water in the theatre [6].

Hospitals across the world affected by drought and water restrictions have begun to explore water waste during scrub procedure and methods to reduce this [7,8]. Waterless alcohol-based hand scrub has also been studied to reduce the environmental impact of surgery [9,10]. Orthopaedic operating theatres are particularly water-intensive, given the high case volumes and the strict need for aseptic conditions when implants are used. Even small savings per scrub add up quickly across busy trauma and elective orthopaedic lists. Sustainability has been highlighted as a growing responsibility within orthopaedics, with reviews pointing to operating theatres as a major contributor to the specialty’s environmental footprint [11,12]. By showing that a modified scrub technique can conserve water without compromising safety, this study provides a practical step towards greener orthopaedic practice and supports broader health system goals of reducing environmental impact.

Aims and objectives

The primary objective of this study was to compare water consumption between the standard continuous flow surgical scrub technique and a modified tap on/off technique. The secondary objective was to compare total scrub duration between the two methods.

With the environmental stresses caused by surgical scrubbing practices, this study is the need of the hour, as this will not only provide us with a rationale for water conservation in orthopaedic theatres but also help in developing guidelines for future surgical scrubbing practices.

## Materials and methods

Operational definitions

Surgical Scrub

A surgical scrub was operationally defined as the thorough cleaning of the hands and forearms for two to five minutes with an antiseptic solution prior to donning sterile gowns and gloves for an operation. Its purpose was to remove dirt, transient microorganisms, and reduce resident skin bacteria, lowering the risk of transmitting pathogens to the patient during surgery.

Standard Technique of Surgical Scrub

In the standard technique, the tap was switched on at the start of the scrub and left running during the whole duration of the surgical scrub. Participants wet their hands and forearms, applied the antiseptic, scrubbed, and rinsed while water continued to flow uninterrupted.

Modified Technique of Surgical Scrub

In the modified technique, the tap was only turned on when water was required, i.e., for the initial wetting step and subsequent rinsing. The tap was turned off while the antiseptic was applied, and the scrubbing was carried out. The antiseptic routine and timing remained unchanged.

Total Scrub Time

This was defined as the full length of the hand scrub, measured from the moment the tap was first turned on to wet the hands until the final rinse was completed. It represented the overall duration of the scrubbing process, regardless of whether the water was running or turned off.

Total Tap-On Time

Total tap-on time referred to the cumulative duration for which the water was actively running during the scrub. In the standard technique, this approximated the total scrub time, while in the modified technique, it only covered the time periods when the tap was turned on for wetting and rinsing.

Study design and setting

This was a within-subject comparative study (quasi-experimental design) where each participant performed two surgical scrubs - one using the continuous tap flow, i.e., standard technique, and another using the intermittent tap (on/off) method, i.e., modified technique. This study was conducted in trauma and orthopaedic operating theatres in Queen Elizabeth Hospital, Birmingham, UK. A total of 34 participants were enrolled in this study. The initial sample size using Stata 15 (StataCorp LLC, College Station, TX, US) indicated the required sample size to be three participants based on the power of the paired means formula using mean 1 = 15.5, mean 2 = 4.5, standard deviation 1 (SD1) = 2.8, SD2 = 1.1, power = 80%, alpha = 0.05, and correlation = 0.2 [7]. We took almost 10 times more participants to cover for variability and dropouts.

The participants were selected using non-probability consecutive sampling, selecting orthopaedic surgical team members (surgeons, nurses, residents) who consented to participate in the study.

The participants included in this study were registered surgical personnel, including surgeons, scrub nurses, and resident doctors who were experienced in standard surgical hand hygiene protocols and consented to participate in the study. No exclusion was made based on the age or gender of the participants. Individuals with skin allergies, wounds, or medical conditions preventing hand scrubbing were excluded from the study, along with the participants who were unwilling to perform both methods of standard and modified surgical scrubbing techniques. As this study was part of a service evaluation, therefore, formal ethical approval was not required. However, this study was registered with the local clinical audit department under the CARMS Code 23180. Consent was taken from all participants before participation.

Data collection method

After obtaining informed consent, each participant performed two surgical scrubs as if preparing for the first case of the day: the standard technique and the modified technique. All participants received standardized instructions to ensure uniformity. For the standard technique, a stopwatch recorded the total scrub duration (from tap opening to closing). For the modified technique, two stopwatches were used. One measured the total scrub time, while the second measured the total tap-on time. All participants used the routinely supplied antiseptic product (chlorhexidine/povidone-iodine) according to local theatre policy.

**Figure 1:**
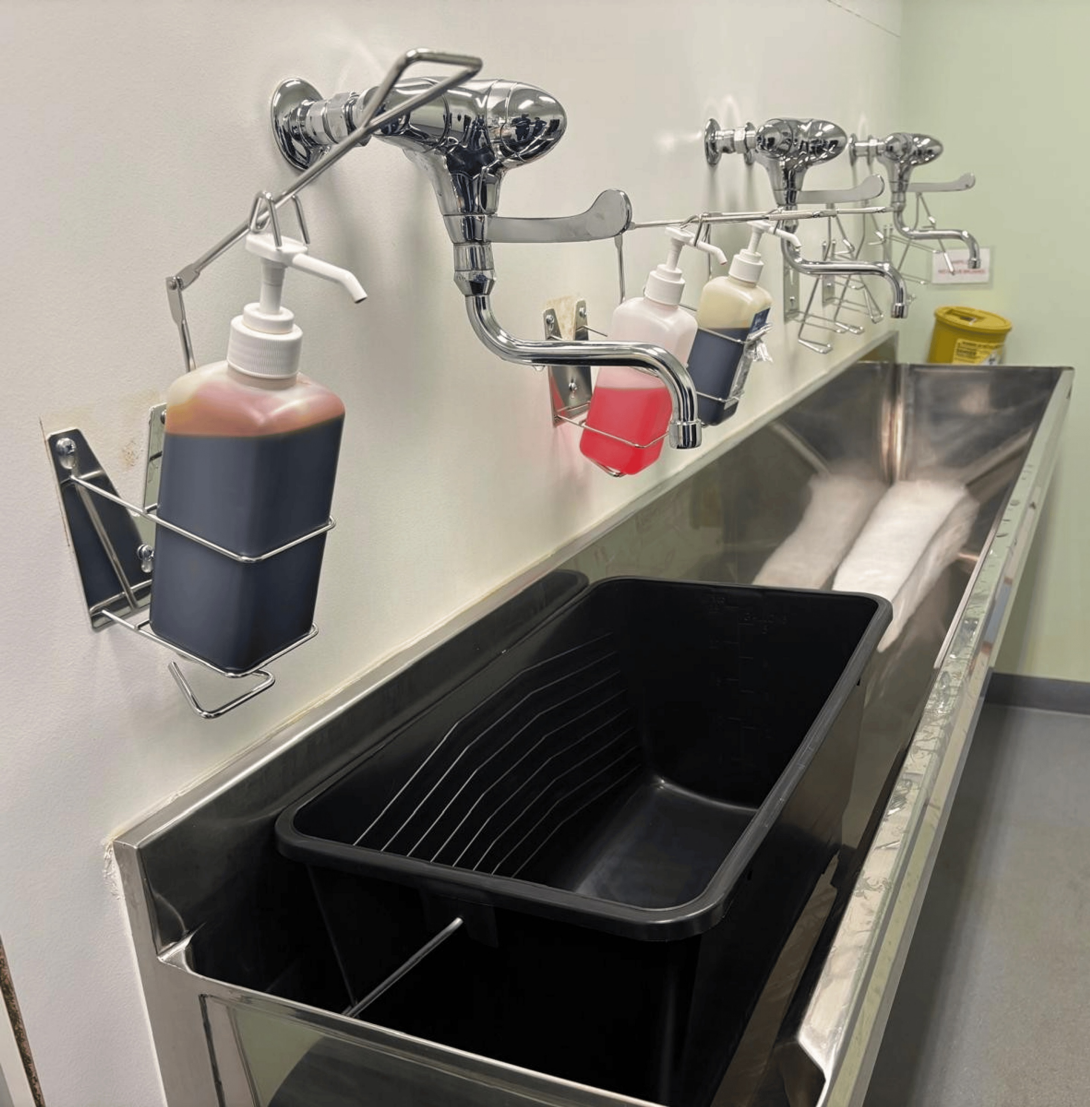
Setup for water collection, showing the 25-L container positioned in the scrub sink.

Water consumption was assessed by collecting all outflow during each surgical scrub in a calibrated 25-litre container placed in the scrub sink. The container was weighed on a digital scale before and after each scrub, with the difference in weight recorded as the total water volume used (1 kg = 1 litre). For each scrubbing technique (standard and modified), the mean water consumption was calculated as the average volume of water used across all participants. The setup created for the collection of water is shown in Figure 1.

Data analysis

Data analysis was done using IBM SPSS Statistics for Windows, Version 25 (Released 2017; IBM Corp., Armonk, New York, United States). Depending on the data obtained, results were compiled and presented. The normality of the data was assessed using the Shapiro-Wilk test. If the data were normally distributed, results were presented as the mean (with SD). If the data were not normally distributed, results were presented as the median (with interquartile range (IQR)).

Quantitative variables such as volume of water used in both techniques, as well as total scrub time and total tap running time, were calculated. The means were compared using a paired t-test, and the significance level was set at p < 0.05. Qualitative variables, i.e., occupational grade of the participant, were represented as percentages and frequencies. Confounders and bias were controlled through strict inclusion and exclusion criteria. All hypothesis testing was performed on paired differences between the two techniques, consistent with a within-subject design.

## Results

A total of 34 healthcare professionals participated in this study, including seven (20.6%) orthopaedic consultants, 15 (44.1%) resident doctors, and 12 (35.3%) scrub nurses. Normality assessment of data was done using the Shapiro-Wilk test. This test demonstrated non-normal distribution for water consumption in the standard technique, while water consumed in the modified technique and the mean water consumed by each participant were normally distributed. Therefore, non-parametric tests, i.e., median (IQR), were applied for water consumption in the standard technique, and parametric tests, i.e., mean ± SD, were used for the modified technique and mean water consumption. The total scrub time for the standard and modified techniques was normally distributed and analyzed using paired t-tests.

Water consumption

There was a substantial reduction in water consumption by participants using the modified technique compared with the standard technique. The median water consumption in the standard technique was 16.2 L (IQR 6.15), while the modified technique recorded a mean ± SD of 5.6 ± 1.78 L. The mean water consumption difference between techniques was 11.34 ± 5.57 L (mean ± SD). This showed that the modified technique reduced water use by approximately 70%. This reduction was statistically significant (p < 0.001), and the calculated effect size was very large (Cohen’s dz = 2.033).

Scrub duration

The mean total scrub time for the standard technique was 2:37.74 ± 00:51.66 minutes (mean ± SD), compared with 2:52.71 ± 00:53.88 minutes (mean ± SD) for the modified technique. The modified technique resulted in a longer scrub duration by 14.96 ± 44.30 seconds on average. However, this increase was not statistically significant (p = 0.057), and the effect size was small to medium (Cohen’s dz = 0.337).

The results are summarized in Table 1.

**Table 1:** Summary of water consumption and total scrub time for the standard and modified techniques IQR: interquartile range

Outcome Measure	Standard Technique	Modified Technique	Mean Difference (Paired)	P-value
Water Consumption (L)	Median 16.2 (IQR 6.15)	Mean 5.6 ± 1.78	11.34 ± 5.57	<0.0001
Total Scrub Time (sec)	Mean 157.74 ± 51.66	Mean 172.71 ± 53.88	+14.96 ± 44.30	0.057
Water Savings (%)	-	-	~70% reduction	-

## Discussion

This study shows that a straightforward alteration to the traditional surgical scrubbing method can lead to a notable decrease in water usage without sacrificing scrub time. The mean water consumption of 34 orthopaedic theatre employees dropped from 16.2 L (IQR 6.15 L) when using the standard technique to 5.6 ± 1.78 L when using the modified technique. This represents a mean savings of 11.34 ± 5.57 L per scrub. Importantly, the total scrub duration did not differ significantly between techniques. These findings affirm that significant water conservation can be attained solely through behavioural modification, in accordance with sustainability objectives within the NHS and worldwide health systems.

The current results are in line with earlier international research showing how much water is wasted during surgical washing and how beneficial intermittent tap use is. According to recent studies, a single traditional scrub requires about 18-21 litres of water [10,13]. Petterwood and Shridhar’s study in the Gold Coast Hospital, Australia, showed that 11 L was saved by using a tap-on/off method, which decreased mean water use from 15.5 L to 4.5 L per scrub [7]. Similarly, at Glasgow's Stobhill Hospital, Somner et al. reported a 5.7 L save with a leg-operated tap [8]. These findings are consistent with the current study's observed decrease of more than 11 L per scrub, which supports the reproducibility of such reductions in various healthcare systems and climates.

While the modified method’s average scrub duration was marginally longer, the difference was statistically insignificant and of limited clinical relevance. This small increase likely reflects the time taken to turn taps on and off rather than any inefficiency in the process. In contrast, the water volume reduction represents a meaningful sustainability gain. During a demanding orthopaedic schedule, frequently surpassing 20 scrub cycles per day in each theatre, this results in an anticipated daily water savings of 220 litres, amounting to more than 80,000 litres annually per theatre.

One of the hospital specialties that uses the greatest resources is orthopaedic surgery, which significantly increases the environmental impact of healthcare. According to earlier studies, operating rooms use a lot of water and energy and can produce up to 30% of hospital waste [13]. Theatres are important targets for resource efficiency measures, according to the NHS's 2020 "Delivering a Net Zero" plan [14]. In this situation, the modified scrubbing method is a practical solution that promotes greener practices right away.

Reducing water use has operational and financial benefits in addition to environmental ones. Hospitals around the world are increasingly impacted by rising utility costs and water scarcity, especially in areas with limited infrastructure or drought. According to Nigerian studies, water shortages caused 5.1% of emergency operations to be delayed [1]. Such hazards can be reduced by using water-efficient scrubbing, which guarantees surgical continuity even in the event of supply disruptions. Additionally, sustainability programs encourage surgical teams to adopt more conscious and beneficial behaviours. Initiatives like single-use reduction, energy saving, and green procurement can be complemented with straightforward, quantifiable behaviours like careful water use, which can act as entry points for more extensive environmental engagement within theatre culture.

Alcohol-based waterless scrubs have also been investigated recently as alternatives to conventional techniques, citing decreases in microbial counts and water consumption. Waterless products achieve comparable antibacterial activity with quicker preparation times and no water usage, according to Rotter [3] and Mastracci et al. [9]. However, issues with skin tolerability, expense, and regulatory acceptability continue to make adoption inconsistent. The modified technique assessed here provides an inexpensive, widely applicable step that significantly reduces water waste while maintaining traditional antiseptic use. This could be a practical intermediate measure before alcohol-based systems are widely used in high-volume or resource-constrained environments.

This study's within-subject design, which reduces inter-individual variance in scrubbing method and duration, is one of its main advantages. Ecological validity was increased by gathering real-world data from orthopaedic practitioners in typical operating room settings. Accurate volumetric evaluation was obtained through objective water measurement using pre- and post-weighing of a calibrated 25-L container.

Nonetheless, several limitations should be considered. As this was a single-centre study using one set of theatre sinks and tap characteristics, generalizability to hospitals with different infrastructure or water pressures may be limited. Differences in water use may be partly influenced by local tap design and flow characteristics, although the paired design ensured internal comparability because each participant used the same tap for both techniques. This study did not include microbiological sampling, bacterial colony counts, or infection-related outcomes; therefore, no conclusions can be drawn regarding antiseptic effectiveness or clinical safety. Water flow length may also be influenced by minor behavioural factors, such as hand posture, the degree to which the tap is turned on, or scrub enthusiasm. Future multi-centre studies that include cost-benefit assessments and microbiological outcomes would strengthen the body of evidence. Additionally, as the participants were aware of being observed, the Hawthorne effect can cause considerable variation in the results as well from routine behaviour.

While feedback systems could offer real-time data on water use, installing motion-sensor or foot-operated taps can further reduce unnecessary flow. A thorough sustainability comparison that considers financial, infection-control, and environmental factors would be made possible by expanding to waterless scrub evaluation. In the end, creating national standards for sustainable surgical hand preparation might guarantee uniform implementation throughout the NHS and beyond.

## Conclusions

This study demonstrates that utilizing the modified technique, the usual surgical scrubbing procedure saves mean water usage by around 70% without significantly altering scrub length. The approach is easy to modify across surgical specialties, straightforward, and cost-neutral. Further research, including microbiological outcomes, would strengthen the clinical safety and effectiveness of the modified scrubbing technique. Adopting this behavioural adjustment shows the orthopaedic community's dedication to providing environmentally responsible care, lowers operating costs, and significantly advances hospital sustainability goals.
